# Foretelling the Future: Preimplantation Genetic Testing and the Coming of Polygenic Embryo Screening

**DOI:** 10.3390/jcm14113885

**Published:** 2025-05-31

**Authors:** Roman Smolarczyk, Anna Szeliga, Anna M. Duszewska, Anna Kostrzak, Ewa Rudnicka, Aleksandra Szczesnowicz, Michał Kunicki, Stefania Bochynska, Gregory Bala, Blazej Meczekalski, Eli Y. Adashi

**Affiliations:** 1 Department of Gynaecological Endocrinology, Medical University of Warsaw, 02-097 Warsaw, Poland; 2 Department of Gynecological Endocrinology, Poznan University of Medical Sciences, 60-179 Poznan, Poland; 3 Department of Morphological Sciences, Faculty of Veterinary Medicine, Warsaw, University of Life Science, 02-787 Warszawa, Poland; 4 UCD School of Medicine, University College Dublin, D04 V1W8 Dublin, Ireland; 5 Department of Medical Science, Brown University, Providence, RI 02903, USA

**Keywords:** PGT, preimplantation genetic testing, polygenic embryo screening, IVF

## Abstract

Preimplantation genetic testing (PGT) has been used in various forms over the last two decades. PGT involves testing early embryos following in vitro fertilization and has now become an accepted part of genetic testing. Nowadays, PGT serves as a resource for couples who have a family history of monogenic disorders, wherein the fetus is at high risk of inheriting the condition. PGT is also used to improve pregnancy outcomes in IVF patients in cases of recurrent IVF implantation failure, recurrent miscarriages, as well as male factor. It is also used in screening for sex-linked disorders and sourcing stem cells for therapy. The latest PGT direction is polygenic embryo screening (PES, PGT-P), which allows the identification of embryos that are at elevated risk for significant diseases in adulthood, such as coronary artery disease (CAD), diabetes, hypertension, and breast cancer. As the prevalence and the potential for the use of PES grow, fundamental ethical issues have been underlined, raising concerns about the broader implications of genetic testing. This narrative review summarizes indications, methods, applications, and limitations for PGT, with a particular focus on PES.

## 1. Introduction

The story of in vitro fertilization (IVF) traces back over a century, marked by numerous trials that have culminated in our present state of practice [1]. The first steps towards developing IVF were in the ground-breaking work on embryo transfer by Walter Heape at the turn of the 19th century. Shortly thereafter, in 1935, Pincus and Enzmann successfully elucidated and documented the chronology of oocyte maturation in rabbits [2].

There are many contributors along the road to developing IVF, amongst whom Robert Geoffrey Edwards holds a significant place. Born in 1925 in Battley, England, Edwards exhibited a profound interest in reproductive immunology, embryology, human chromosomes, and embryo transplantation in women [3].

Prior to his most notable achievements, R. G. Edwards studied egg maturation, aiming to understand the mechanisms leading to chromosomal abnormalities such as Turner’s, Down’s, and Klinefelter’s syndromes. In 1968, Edwards met English physician Patrick Steptoe, and the two embarked on a novel research initiative focused on extracting mature human oocytes and conducting trial fertilization [4]. Over two years (1971–1972), Patrick Steptoe and R. G. Edwards conducted their first embryo transfers (ETs), using ovarian stimulants and hCG. This endeavor counted 150 laparoscopic oocyte recoveries (LORs) and resulted in the first, albeit ectopic, pregnancy in 1976.

A historic milestone in fertility medicine was achieved on 25 July 1978, with the birth of the first in vitro baby, Louise Brown. Her delivery was via cesarean section, led by Patrick Steptoe with assistance from John Webster [5]. Another pioneer in reproductive medicine was Howard W. Jones Jr., born in 1910 in Baltimore. After training in general surgery, Jones developed an interest in vitro fertilization and in 1965 collaborated with Robert Edwards on the first fertilization of a human egg outside the body [6]. In recognition of his pioneering contributions, R. G. Edwards was awarded the 2010 Nobel Prize in physiology and medicine for his work on in vitro fertilization [7].

IVF paved the way for preimplantation genetic testing (PGT), which has been used in various forms over the last two decades [8]. This procedure involves testing early embryos following in vitro fertilization and has now become an accepted part of genetic testing. In the late 1980s, this technique was developed in the United Kingdom as a measure to prevent the transmission of adrenoleukodystrophy and X-linked mental retardation [9]. In the contemporary landscape, PGT is commonly used to screen for autosomal dominant, autosomal recessive, and X-linked abnormalities. Notably, chromosome aneuploidy stands as one of the leading causes of pregnancy loss, exhibiting a higher incidence among women aged 35 and above. PGT has confirmed the high incidence of aneuploidy in gametes and embryos [10]. PGT serves as a resource for couples who have a family history of single-gene disorders, wherein the fetus is at high risk of inheriting the condition. A new tool in preimplantation genetic testing is polygenic embryo screening (PES, PGT-P) based primarily on genome-wide association studies (GWAS) with their modifications [11].

## 2. Selected Trends Preimplantation Genetic Testing (PGT): Indications and Diagnostic Methods

Essentially, preimplantation genetic testing (PGT) encompasses three distinct types: PGT for monogenic (single-gene) disorders (PGT-M), PGT for structural rearrangements (PGT-SR), and PGT for aneuploidy (PGT-A), but it is also worth mentioning non-invasive preimplantation genetic testing (NiPGT). Each of these techniques carries specific indications (Table 1).

### 2.1. Preimplantation Genetic Testing for Monogenic (Single-Gene) Disorders (PGT-M)

- Patients identified to be at increased risk of having offspring with medically actionable conditions:

PGT-M serves to establish pregnancies that are unlikely to be affected by certain genetic conditions to which the children are predisposed. Examples of these conditions include cystic fibrosis, sickle cell disease, spinal muscular atrophy, Duchenne muscular dystrophy, and hemophilia. For patients who would prefer to discard affected preimplantation embryos or oocytes rather than later undergo the termination of an affected pregnancy, this procedure is of great significance. When adequately informed, most couples tend to prefer PGT over prenatal diagnosis (PND) [12].

The American Society for Reproductive Medicine has expressed that PGT-M for adult-onset conditions is: “ethically justifiable when the conditions are serious and when there are no known interventions for the conditions or the available interventions are either inadequately effective or significantly burdensome”. In cases of conditions that are less severe or have lower penetrance, PGT-M for adult-onset conditions is deemed ethically acceptable, upholding the principle of reproductive liberty. An experienced genetic counselor must guide patients who contemplate such procedures [13].

- Patients seeking HLA-compatible siblings for stem cell therapy:

For individuals desiring to conceive a child whose Human Leukocyte Antigen (HLA) type is compatible with that of a sibling, PGT with HLA matching is of crucial importance. This process aims to secure a disease-free embryo that is not only compatible with the affected sibling’s HLA type but also capable of providing cord blood stem cells for transplantation. The selection of an unaffected embryo with HLA compatibility significantly improves the survival prospects of the afflicted sibling. However, an inherent limitation of this strategy is that only a relatively small percentage (approximately 16 percent) of tested embryos will simultaneously exhibit the absence of ailment and HLA compatibility for the affected sibling [14].

- Patients aiming to avoid sex-linked disorders:

PGT-M finds application in cases where individuals wish to prevent the inheritance of sex-linked disorders in their offspring. This method identifies embryos that are either unaffected males or non-carrier females, enabling a preference for transferring these embryos into the uterus. The most common indications for such testing frequently involve disorders like Duchenne muscular dystrophy and hemophilia [15].

- Patients endeavoring to prevent the passing of suspected autosomal dominant disorder:

PGT can assess embryos for autosomal dominant diseases with late-onset manifestations, such as Huntington’s chorea. This is particularly important, as patients become aware of their disease only after a close relative is diagnosed with the condition. In these circumstances, patients are usually aware of their 50 percent likelihood of carrying the pathogenic variant. Given this information’s personal and often also insurance implications, many individuals opt not to ascertain their carrier status for the variant. In such cases, PGT can be performed without disclosing the conclusive outcome, should the potentially affected parent remain uninformed about the test result [15,16].

### 2.2. Preimplantation Genetic Testing for Structural Rearrangements (PGT-SR)

- Patients with an established diagnosis of an existing balanced translocation:

PGT for structural rearrangements addresses the presence of a proven balanced translocation in one of the partners. This method has a demonstrated substantial reduction in the frequency of miscarriage among couples experiencing recurrent pregnancy loss, attributed to the existence of an identifiable balanced translocation in one partner [17].

### 2.3. Preimplantation Genetic Testing for Aneuploidy (PGT-A)

- Aging patients who desire elective single-embryo transfer (eSET):

In cases where aging patients prefer elective single-embryo transfer (eSET), several studies have demonstrated higher birth rates following aneuploidy testing and eSET. This suggests the potential of aneuploidy testing in mitigating the risk of multiple gestations, despite notable limitations in these studies [13].

Older patients, particularly those ≥ 37 years of age, bear an increased risk of producing aneuploid embryos. Therefore, applying PGT-A in this demographic can facilitate the selection of euploid embryos for eSET, thereby increasing the efficacy (live birth rate per transferred embryo) of in vitro fertilization. Research has observed a marked increase in ongoing pregnancy rates per embryo transfer through the integration of PGT, specifically observed within the subset of women aged 35–40 who possess two or more embryos amenable to biopsy [18].

An additional study conducted by Cheng X. et al. outlined that PGT-A did not universally lead to an elevated live-birth rate [19]. They observed that PGT-A positively influences live-birth rates in women of advanced maternal age but not among those in younger age groups [19].

Preimplantation genetic testing for aneuploidy has progressed to include the assessment of all chromosomes, employing various techniques such as array comparative genomic hybridization and next-generation sequencing [20,21].

### 2.4. Non-Invasive Preimplantation Genetic Testing (niPGT)

NiPGT is a method of analyzing embryos for genetic abnormalities without the need for a biopsy. It involves collecting cell-free DNA from the embryo’s culture medium and analyzing it to identify chromosomal imbalances like aneuploidy. This technique offers a less invasive alternative to traditional PGT-A, which involves taking a biopsy of the embryo.

Non-invasive Preimplantation Genetic Testing for Aneuploidy (niPGT-A) involves testing material taken from the embryo’s culture medium at an early stage of its development. Unlike PGT-A, in which only trophectoderm cells are used, niPGT-A reflects the ploidy state of these cells and internal cell mass, suggesting that this technology may be less prone to error, being more reliable than the invasive test [22].

## 3. Polygenic Embryo Screening (PES, PGT-P) as a New Technique of Preimplantation Genetic Testing: Applications and Limitations

Polygenic Embryo Screening is primarily based on genome-wide association studies (GWAS), which enable the identification of genetic variations or variants linked with specific traits or diseases [23]. These studies have led to the creation of polygenic risk scores (PRSs), amalgamating numerous genetic variants (individually exhibiting small effects) into a single risk estimate [24]. PRSs can be used to identify embryos selected for in vitro fertilization (IVF) that are at elevated risk for significant diseases in adulthood, such as coronary artery disease, diabetes, hypertension, and breast cancer [11]. Traditionally, preimplantation genetic testing was primarily sought by couples with a family history of genetic disorders such as Huntington’s and Tay-Sachs disease [24].

Studies show that individuals with very high PRSs generally exhibit incidence rates significantly surpassing the population average [11]. Within the polygenic embryo screening (PES) framework, employing a prioritization strategy favoring the implantation of embryos with the lowest PRS can yield substantial relative risk reductions for diseases. While excluding high-risk embryos yields moderate risk reduction, the embryos with the lowest PRS can yield substantial relative risk reductions [25].

The nascent adoption of PES introduces the prospect for individuals or couples who might not have otherwise considered IVF to do so to capitalize on the service. For some prospective parents, opting for PES may be regarded as an informed and responsible reproductive choice [26]. The initial implementation of Preimplantation Genetic Testing for Polygenic Disease (PGT-P) may be particularly well suited to those in the population suffering from infertility, given their increased risk of cardiovascular disease, cancer, and diabetes. The incorporation of polygenic embryo screening into clinical applications may provide a means to reduce the prevalence of disease in humans [27].

However, recent progress in complex trait genetics, which paved the way for genetic embryo screening for polygenic traits, has not escaped controversy [11]. According to Karavani et al., embryo screening for polygenic traits presents limited utility when considering the scientific, practical, and ethical aspects [28]. Simulations, models, and empirical data collectively show that the gain in trait value when selecting the top-scoring embryo remains limited and uncertain [29].

In 2022, the Executive Committee of the European Society of Human Genetics (ESHG) published a critical paper concerning polygenic risk scores in preimplantation genetic testing [30]. Unfortunately, no clinical research has to date been published comprehensively evaluating the effectiveness of this strategy. Patient awareness regarding the limitations of this procedure is paramount. The use of PRS should be considered concomitantly with the assessment of additional factors such as lifestyle, nutrition, physical activity, and environment [30]. According to the European Society of Human Reproduction and Embryology (ESHRE) [31] and also the American Society for Reproductive Medicine (ASRM), ESHG, and the American College of Medical Genetics (ACMG), concerns regarding the fundamental use of PES exist and need to be addressed. First, the genetic testing pool in any IVF cycle is invariably limited, thereby rendering each embryo predisposed to heightened PRS for specific traits or diseases. Consequently, the mere exclusion of embryos with exceedingly high PRSs cannot yield a substantive risk reduction. According to the above organizations, PES fundamentally differs from the rationale in genetic testing for monogenic diseases, wherein only affected (or very high-risk) embryos are deselected to prevent substantial and probable harm. The second concern underscores the considerable overlap in a sibling cohort of embryos evaluated by PRS, which significantly limits its predictive value. Ultimately, an array of minor risk factors will be exhibited and will overlap and be traced across a many gene variants inherited from parental genes, especially for many traits and pedigrees. The third concern underscores the inability of PRS to incorporate phenotypic or environmental data, precluding a reliable risk estimate for complex diseases. Finally, the intricate interactions between distinct genetic variants remain poorly understood, presenting a scenario where embryo selection aimed at safeguarding against one disease might inadvertently increase the risk for others.

## 4. Polygenic Risk Scores (PRS): Clinical Applications and Challenges

PRS assessment offers valuable insight into the likelihood of developing polygenic diseases, playing a significant role in reproductive medicine. However, alongside its advantages, PRS also has its inherent limitations. One primary challenge is that PRS scores do not provide an absolute predictor of disease risk, [32,33,34,35,36,37,38]. Nonetheless, several statistical tools have been developed, aimed at improving the predictive power of PRS [32,33].

Currently, PRS is used to compute an array of conditions, including breast cancer, coronary artery disease, diabetes, schizophrenia, bipolar disorder, and Alzheimer’s disease, among others [25,38,39,40,41,42,43,44]. Given the varying degrees of influence environmental factors have on each polygenic disease, the methodology and tools for PRS estimation vary widely. The distinct approaches to PRS estimation can be traced when analyzing diseases such as breast cancer, coronary artery disease, and adverse drug reactions (ADR).

Epidemiological data show that breast cancer is the most prevalent cancer globally [45]. Statistical models such as the Gail Model Breast and Ovarian Analysis of Disease Incidence and Carrier Estimation Algorithm (BOADICEA) consider established breast cancer risk factors, such as personal and family history, lifestyle factors, and breast histopathology [46,47]. Various studies suggest that PRS serves as a strong predictor of breast cancer risk, with a more than twofold difference in risk between the lowest and highest PRS quartiles. Despite some limitations, extensive studies such as the PROCAS and WISDOM trials have explored the impact of incorporating PRS into breast screening practices [48,49].

Coronary artery disease, a leading global cause of mortality [50], offers a different perspective. Studies present conflicting data regarding the clinical utility of PRS in this context. While some research has shown improvements in risk prediction accuracy compared to conventional risk factors, others report only marginal benefits. A primary limitation in these studies includes limited participant diversity, mainly of European origin [34,51,52,53,54]. Moreover, some studies suggest that the PRS predictive ability for CAD was more reliable in younger people [55].

Pharmacogenetic studies also leverage PRS [56] to identify genetic variants influencing treatment response or rare variants that may predispose the carrier to an increased risk of adverse drug reactions (ADR) [57]. These studies aim to evaluate the interplay between genetics and drug response, aiding in selecting the most appropriate therapeutic option for individual patients. An illustrative example involves a hypersensitivity reaction to abacavir, strongly associated with the presence of the HLA-B*5701 allele [58]. Studies that examined the treatment of psychiatric, circulatory, and digestive disorders identified 23 phenotypes related to ADR and 82 linked to drug efficacy and treatment response [59]. Notably, investigations have focused on PRS in conjunction with commonly prescribed drugs such as antipsychotics, anticoagulants, and statins in the context of efficacy and ADRs [60]. However, challenges in PRS and pharmacogenetics necessitate uniform patient treatment and well-defined endpoints, all while factoring in the ever more commonplace cases of complex polypharmacy, which increase the risk of drug–drug interactions.

It is worth considering the utility of PRS in clinical practice when factoring in the time discrepancy between testing and disease probability, as well as the severity of environmental factors. Many facets need to be weighed when considering PRS for embryo selection [40,41].

## 5. The Growing Commercialization of Polygenic Embryo Screening (PES, PGT-P) and Relevant Legal Frameworks

The first clinical case involving polygenic risk scoring for type 1 diabetes in human preimplantation embryos was introduced by Genomic Prediction (LifeView) and reported in 2019 by Treff et al. [61] Genomic Prediction has developed technologies enabling concurrent screening for a multitude of conditions, including type 2 diabetes, schizophrenia, coronary artery disease, breast cancer, prostate cancer, inflammatory bowel disease, Alzheimer’s disease, and idiopathic short stature. Furthermore, Genomic Prediction offers embryo testing for intellectual disability [27,62,63,64]. According to public data provided by the company (lifeview.com), in 2020, Genomic Prediction operated across six continents, with a presence in 150 clinics worldwide.

In addition to Genomic Prediction (LifeView), several other commercial enterprises offer PGT-P services, including Orchid Health (orchidhealth.com), MyOme (myome.com), Virginia Center (vcrm.com), and Reprocare Genetics (reprocaregenetics.com). These entities offer PES for disease traits (cancer, diabetes, heart disease, schizophrenia, Alzheimer’s disease, among others) and non-disease traits such as height and IQ [61,64,65,66].

It is worth noting that these companies actively engage in discussions regarding the ethical aspects of PES, and their associates frequently participate as co-authors in scientific publications on polygenic embryo screening. These publications often address the implementation as well as the ethical aspects of PES (Genetics Prediction [61], Orchid Health [67], MyOme [29]).

Companies offering PES only provide embryo selection but refrain from verifying this selection due to the difficulty in extrapolating results from polygenic screening in adults to individuals already affected by a disease, mainly when based on Western populations. Furthermore, the incidence of many diseases correlates with age (e.g., prostate cancer, coronary artery disease, Alzheimer’s, among others) and is often accompanied by comorbidities. Consequently, whether individuals subjected to PES will be subject to corporate scrutiny, at the behest of either their parents or themselves, for the verification and correlation of the effectiveness of embryo selection remains unclear.

While PGT-A, PGT-M, and PGT-S procedures are generally accepted and regulated worldwide, albeit with some exceptions [65,68], the PES procedure continues to await judicial testing in most jurisdictions. Nevertheless, PES-IVF is available in the United States, where a liberal stance towards the regulation of assisted reproductive technologies prevails [62]. In contrast, countries such as the United Kingdom, Australia, Italy, Switzerland, and France have yet to establish regulatory frameworks for PES, a scenario also mirrored in many other countries. Nonetheless, discussions concerning legislation have been ongoing in Europe for many years [69], further necessitating global debate. Several initiatives have been launched towards this end [70], with the most notable being the creation of an initial framework on which to consider the ethical, legal, and social implications (ELSI) of PES by the Polygenic Embryo ELSI Research Group (polygenicembryo.org).

## 6. Ethical Considerations in Polygenic Embryo Screening (PES, PGT-P)

While the preimplantation screening of embryos for aneuploidy (PGT-A), chromosomal aberrations (PGT-S), and monogenic diseases (PGT-M) is widely accepted [40,71], the use of polygenic embryo screening (PES) remains immersed in controversy within both the scientific community and the media (polygenicembryo.org). It is essential to know that PES is still in the experimental phase and that most data comes from modeling rather than clinical outcomes. Hence, profound reservations exist regarding the use of polygenic screening to select human embryos based on polygenic disease or non-disease traits [26,27,41,61,65,66,72,73,74,75].

Many researchers have extensively covered the fundamental ethical issues related to PES [11,76,77,78]. We believe that the ethical aspects of PES should include the representativeness of polygenic screening results, embryo selection, and social ethics.

Polygenic embryo screening relies on a method known as genome-wide association study (GWAS), which identifies a large number of genetic variants, typically single-nucleotide polymorphisms (SNPs), associated with a wide array of complex traits (genome.gov/genetics-glossary/Genome-Wide-Association-Studies (accessed on 28 May 2025)) [32,69]. This analysis estimates the risk of a polygenic trait (polygenic risk score or PRS) rather than a diagnostic outcome [61]. In such a context, PRS introduces a potential conflict between research ethics and assessment ethics. While PRS exhibits significant reliability for diseases such as type 1 or type 2 diabetes, hypertension, and breast cancer, its reliability diminishes for psychiatric disorders such as schizophrenia, dementia, and many others [11,25,28,66,79]. Furthermore, the efficacy of PES for diseases is contingent on the selection strategy due to the intricate nature of polygenic traits [25], characterized by gene–gene interactions (including pleiotropy), and varying degrees of influence from environmental factors [35,64,70,80]. Ethical concerns regarding the reliability of this method revolve around the representativeness of the PRS itself. As a value, it may be limited by variables such as a couple’s genetic makeup, race, parental history, gender, family history, continent of residence, and more [32]. A broad application of PES for numerous traits could result in the exclusion of nearly all embryos, which may not align with the intended goals of the provider and the client.

Furthermore, PES often leads to complicated and often ethically dynamic relationships between geneticists, physicians, and parents, as described extensively in many scientific journals and media reports [26,65]. Legitimate concerns arise regarding the potential for PES to interfere with parent–child relationships, particularly in the context of selecting non-disease traits [70]. While parents have the ultimate say in embryo selection, medical professionals, including doctors and geneticists, must be cognizant of their involvement in the decision-making process and its ensuing consequences.

Social ethics pertain to a systematic examination of the morality linked to the social ramifications of PES. While PES offers specific positive social implications, it also presents many challenges. These range from the creation of a genetically homogenous population, the pressure to supplant natural reproduction with PES-IVF, and concerns regarding equal access to PES and, consequently, to assisted reproduction. This implies that some individuals may opt for IVF solely to prevent polygenic disorders rather than to address infertility. Furthermore, inequality of access, societal stratification, significant demographic shifts, and skewed sex ratios in a population come into play [41]. The social implications precipitate ethical concerns, including discrimination, glorification, stigmatization, and many other issues discussed in various scholarly works [11,65,70].

While polygenic screening in adults undoubtedly opens new avenues for understanding the etiology of many diseases and assessing traits, the ethical standpoint suggests that using the polygenic screening of embryos to forestall polygenic diseases may be a premature and precipitous step. Genetic testing is destined to advance continuously, paralleling the evolution of medicine. This synchronicity holds promise for the development of pioneering approaches to prevent and treat a multitude of polygenic disorders. Employing polygenic screening to forestall polygenic diseases ultimately stunts this development.

A separate issue of concern is employing PES for non-disease traits. PES exclusively focuses on human genetic selection, disregarding natural selection, and does not account for pleiotropy and environmental factors. Consequently, PES is a technique that sieves out diversity and normal variations of potential benefit to our population.

## 7. Conclusions

The preimplantation genetic testing of embryos has opened a new chapter in infertility treatment as diagnostic (PGT-A; PGT-SR; PGT-M; niPGT) and predictive [PGT-P(PES)] tools. As a diagnostic tool, PGT enables the detection of genetic defects or mutations. Therefore, it is a representative marker commonly used in IVF programs. In contrast, PGT-P as a predictive tool only determines the risk of polygenic diseases (PRS), including multigene and multifactorial (genetics–epigenetics–environment) diseases. PGT-P is still in the research phase, and its clinical application raises questions about the representativeness of PRS and many ethical dilemmas.

## Figures and Tables

**Table 1:** PGT-A unsubstantiated indications table.

Indication	Explanation/Context	Role of PGT-A
IVF outcomes in general	PGT-A proposed to improve live birth and miscarriage rates.	No clear evidence for benefit in live birth or miscarriage rates.
Recurrent IVF implantation failure	Embryos may look normal but carry chromosomal abnormalities.	Helps select embryos with normal chromosomes, especially after 3 failed transfers.
Recurrent miscarriages	~50% due to embryo chromosome abnormalities.	Helps select embryos free from structural or numeric chromosomal issues.
Male factor infertility	Male factor is a major cause of embryonic aneuploidy.	Enables selection of embryos with chromosomal integrity.
